# Withdrawal from Extended, Intermittent Access to A Highly Palatable Diet Impairs Hippocampal Memory Function and Neurogenesis: Effects of Memantine

**DOI:** 10.3390/nu12051520

**Published:** 2020-05-23

**Authors:** Antonio Ferragud, Clara Velázquez-Sánchez, Ali Al Abdullatif, Valentina Sabino, Pietro Cottone

**Affiliations:** Laboratory of Addictive Disorders, Departments of Pharmacology and Psychiatry, Boston University School of Medicine, Boston, MA 02118, USA; af626@cam.ac.uk (A.F.); cv326@cam.ac.uk (C.V.-S.); aliha@mit.edu (A.A.A.); vsabino@bu.edu (V.S.)

**Keywords:** compulsive eating, neurogenesis, NMDA

## Abstract

Background: Compulsive eating can be promoted by intermittent access to palatable food and is often accompanied by cognitive deficits and reduction in hippocampal plasticity. Here, we investigated the effects of intermittent access to palatable food on hippocampal function and neurogenesis. Methods: Male Wistar rats were either fed chow for 7 days/week (Chow/Chow group), or fed chow intermittently for 5 days/week followed by a palatable diet for 2 days/week (Chow/Palatable group). Hippocampal function and neurogenesis were assessed either during withdrawal or following renewed access to palatable food. Furthermore, the ability of the uncompetitive *N*-methyl-d-aspartate receptor (NMDAR) antagonist memantine to prevent the diet-induced memory deficits and block the maladaptive feeding was tested. Results: Palatable food withdrawn Chow/Palatable rats showed both a weakened ability for contextual spatial processing and a bias in their preference for a “novel cue” over a “novel place,” compared to controls. They also showed reduced expression of immature neurons in the dentate gyrus of the hippocampus as well as a withdrawal-dependent decrease of proliferating cells. Memantine treatment was able both to reverse the memory deficits and to reduce the excessive intake of palatable diet and the withdrawal-induced hypophagia in food cycling rats. Conclusions: In summary, our results provide evidence that withdrawal from highly palatable food produces NMDAR-dependent deficits in hippocampal function and a reduction in hippocampal neurogenesis.

## 1. Introduction

Both the increased availability of energy-dense, highly palatable foods and dieting are believed to contribute to the development of obesity and certain forms of eating disorders [1,2]. Highly palatable food drives overeating often followed by dieting, which is culturally driven by norms for thinness or health [3]. Similar to what is observed in drug addiction, abstaining from highly palatable foods is hypothesized to contribute to the emergence of a negative emotional state, which in turn sustains cravings and promotes relapse, resulting in a vicious circle of palatable food withdrawal and compulsive eating [4].

Individuals affected by forms of eating disorders and obesity have been shown to display impairments in multiple domains of cognition, including hippocampal-dependent memory function [5,6,7]. The hippocampus is an important brain area that plays a key role not only in contextual learning and memory processes [8], but also in feeding behavior [9], as well as affective and addiction disorders [10,11]. One prominent feature of hippocampal plasticity is adult neurogenesis, the ability of the dentate gyrus subgranular zone (SGZ) to give rise to new neurons throughout life [12]. Accumulating evidence suggests that neurogenesis plays a pivotal role in affective as well as addictive disorders [10,13,14].

A series of experiments was performed to evaluate the effects of alternation of palatable food withdrawal and refeeding on memory function and hippocampal neurogenesis. The first aim of this study was to assess whether withdrawal from chronic, intermittent access to a highly palatable diet impairs memory function. For this purpose, we used a battery of behavioral tasks not involving appetitive reinforcers or food restriction/deprivation to evaluate different aspects of memory function following an established rodent model of compulsive eating induced by palatable food cycling [15,16]. The second aim of the study was to assess whether withdrawal from chronic, intermittent access to a highly palatable diet impairs key neurogenic processes, such as the number of proliferating cells and the number of newborn neurons in the dentate gyrus SGZ of the hippocampus. Furthermore, the third aim of this study was to evaluate the effects of the uncompetitive *N*-methyl-d-aspartate receptor (NMDAR) uncompetitive antagonist memantine, a pro-cognitive drug used for the treatment of Alzheimer’s disease, on palatable food withdrawal-induced memory dysfunction. Lastly, the fourth aim of this study was to evaluate the effects of memantine on food intake during palatable food withdrawal and refeeding.

## 2. Materials and Methods

### 2.1. Subjects

Male Wistar rats, weighing 180–230 g and 41–45 days old on arrival (Charles River, Wilmington, MA, USA), were single-housed in wire-topped plastic cages (27 × 48 × 20 cm) in a 12-h reverse light cycle (lights off at 11:00 h), in an AAALAC-approved humidity- and temperature-controlled vivarium. Upon arrival, rats had ad libitum access to corn-based chow (Harlan Teklad LM-485 Diet 7012 (65% (kcal) carbohydrate, 13% fat, 21% protein, 341 Kcal/100 g); Harlan, Indianapolis, IN, USA) and water, otherwise specified. Procedures adhered to the National Institutes of Health Guide for the Care and Use of Laboratory Animals (NIH publication number 85-23, revised 1996) and the Principles of Laboratory Animal Care (http://www.nap.edu/readingroom/bookslabrats), and were approved by the Boston University Institutional Animal Care and Use Committee (IACUC). No experimental procedures involved food or water restriction/deprivation.

### 2.2. Drugs 

Memantine hydrochloride (1,3-dimethyl-5-aminoadamantane hydrochloride) was purchased from Acros Organics (Geel, Belgium). Memantine was dissolved in isotonic saline immediately before administration. Memantine was administered intraperitoneally (i.p.), using randomized within-subject Latin square designs, at doses of 0, 2.5, 5, and 10 mg/kg for the feeding studies, and in between-subjects designs at the doses of 0 and 10 mg/kg for the memory tasks. Memantine was administered in rats diet-cycled for at least seven cycles either upon renewing access to the palatable diet (C→P phase) or to the chow diet (P→C phase). The pretreatment time was 30 min. Doses, injection volume, and pretreatment times were based on previously published reports [17,18,19].

### 2.3. Ad libitum Diet Alternation

**Figure 1:**
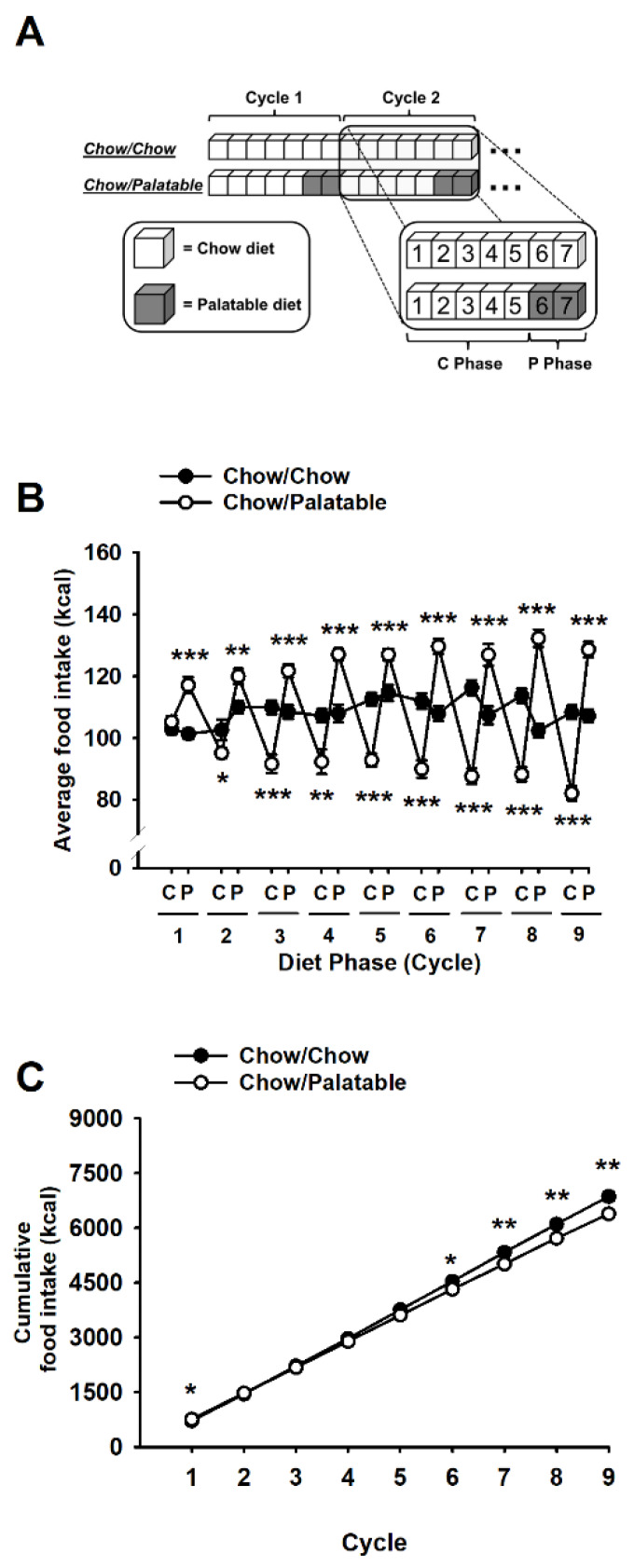
(**A**) Schematic representation of the experimental design used in this study. Animals were divided in two groups: one received a standard chow diet daily (Chow/Chow group) and the other group received the same standard chow for 5 days followed by a highly palatable chocolate-flavored, high sucrose diet for 2 days (Chow/Palatable group). The first 5 days when chow diet was available and the last 2 days of the weeks (when animals received either chow or palatable depending of the experimental conditions) of each week were referred as C and P phases. (**B**,**C**) Effects of repeated, alternating 5-day access to chow and 2-day access to either regular chow (Chow/Chow) or a palatable diet (Chow/Palatable) in male Wistar rats on (B) local and (C) cumulative food intake (*n* = 19–20/group). Data show *M* ± SEM. * *p* ≤ 0.05, ** *p* ≤ 0.01, *** *p* ≤ 0.001 vs. Chow/Chow.

Ad libitum diet alternation was performed as previously described [20,21,22]. After acclimation, rats were divided into two groups matched for food intake, body weight, and feed efficiency from the previous 4 days. One group (Chow/Chow) was provided a chow diet (Harlan Teklad LM-485 Diet 7012) ad libitum 7 days a week, while a second group (Chow/Palatable) was provided chow ad libitum 5 days a week followed by 2 days of ad libitum access to a highly palatable, chocolate flavored, high-sucrose diet. The palatable diet was a nutritionally complete, high-sucrose (50% kcal), AIN-76A-based diet that is comparable in macronutrient proportions and energy density to the chow diet (5TUL: 66.7% (kcal) carbohydrate, 12.7% fat, 20.6% protein, 344 kcal/100 g; TestDiet). For brevity, the first 5 days (chow only) and last 2 days (chow or palatable according to experimental group) of each week are referred to as C and P phases in all experiments (Figure 1A). Palatable diet was provided in the home cage in GPF20 ‘J’-feeders (Ancare, Bellmore, NY, USA). Rats were provided with pre-weighed food at dark cycle onset. Food intake and body weight were measured at the beginning of the C and P phases throughout the whole experiment; diets were never concurrently available [15,23]. All rats were diet cycled for at least 7 weeks before any behavioral or pharmacological testing.

### 2.4. Object Location (OL) Task

**Figure 2:**
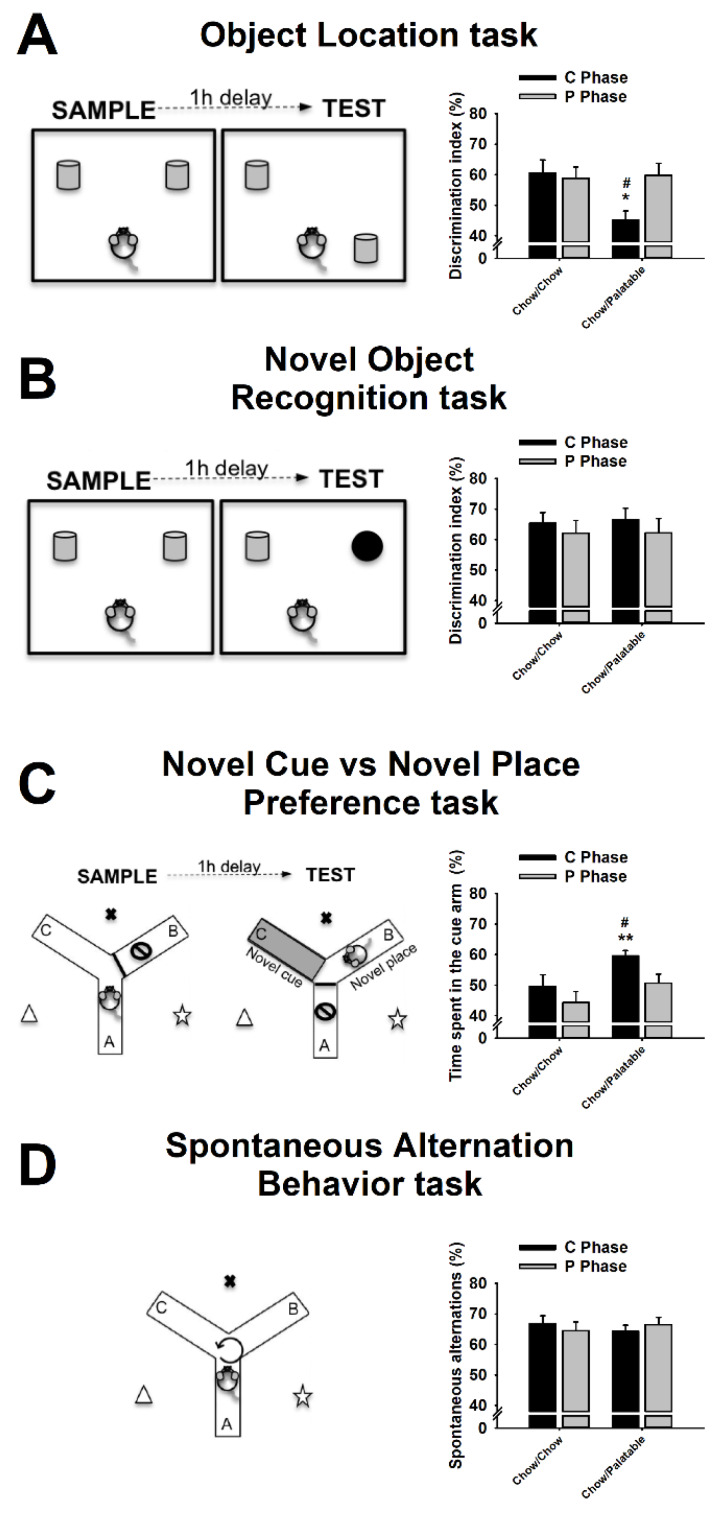
Effects of repeated, alternating 5-day access to chow and 2-day access to either regular chow (Chow/Chow) or a palatable diet (Chow/Palatable) in male Wistar rats on (**A**) Object Place Recognition task; (**B**) Novel Cue vs. Novel Place Preference task; (**C**) Spontaneous alternation task; (**D**) Object Recognition task (*n* =8–10/group). Data show *M*±SEM. # *p* ≤ 0.05, Chow/Palatable in C phase vs. Chow/Palatable in P phase; * *p* ≤ 0.05, ** *p* ≤ 0.01 Chow/Palatable in C phase vs. Chow/Chow in C phase.

The Object Location (OL) task probes spatial memory and consists in the exploration of an object, which has been moved from a familiar to a novel position [24]. Rats were habituated for 10 min to the apparatus (50 × 70 × 35 cm arena) the day before testing. Testing occurred either on day 1 (P→C) or on day 6 (C→P), 4 to 8 h after food switch. The task consisted of two trials: a sample trial and a test trial (Figure 2A, left panel). During the sample trial, rats were allowed to explore two identical objects located on two adjacent corners of the arena, for 5 min. After 1 h, a 5 min test trial was performed, in which one of the two objects was moved to a novel position. The objects were made of glass and ceramic (Dollar Tree, Boston, MA). The object exploration time was defined as the time the rat spent with its nose oriented towards and within 2 cm to the object. The discrimination index percentage was used as the dependent variable and was calculated as [time spent exploring the object in the novel position/total exploration time] × 100 [25]. Rats typically spend more time exploring the object in the novel position, and a reduction of this tendency is interpreted as a deficit in spatial memory [25].

### 2.5. Novel Object Recognition (NOR) Task

The Novel Object Recognition (NOR) task is used to evaluate recognition memory and is based on the spontaneous tendency of rats to spend more time exploring a novel object than a familiar one [26]. Rats were habituated for 10 min to the apparatus (50 × 70 × 35 cm arena) the day before testing. Testing occurred either on day 1 (P→C) or on day 6 (C→P), 4 to 8 h after switching food, in rats food cycled for at least 7 weeks. The task consisted of two trials: a sample trial and a test trial (Figure 2B, left panel). During the sample trial, rats were allowed to explore two identical objects located on two adjacent corners of the arena for 5 min. After 1 h, a 5 min test trial was performed, in which a familiar object (an object that was identical in shape and color to the ones used in the sample trial, but not exactly the same to prevent unwanted olfactory cues) was presented concurrently with a novel object (an object that was different in shape and color from the ones used in the sample trial). The objects were made of glass and ceramic (Dollar Tree, Boston, MA, USA) and varied in color, shape, and dimension. Familiar and novel objects were counterbalanced between animals. The object exploration time was defined as the time the rat spent with its nose oriented towards and within 2 cm to the object. The discrimination index % was used as the dependent variable and was calculated as [time spent exploring the novel object/total exploration time] [25]. Rats typically spend more time exploring the novel object, and a reduction of this tendency is interpreted as deficit in recognition memory.

### 2.6. Novel Cue vs. Novel Place Preference (NC-NP Preference) Task

This Novel Cue vs. Novel Place (NC-NP) preference task is used to assess a subject’s preferential expression of stimulus-response vs. spatial learning [27,28,29]. The apparatus consisted of a Y-shaped maze with three white, enclosed, opaque plastic arms (7.6 × 45.8 cm) at a 120° angle from each other (Med Associates Inc, Fairfax, VT, USA) (Figure 2C, left panel). The color of the floors of the three arms was changed (white or blue) by using plastic inserts (Med Associates Inc, Fairfax, VT, USA). Access to arms was prevented by using removable doors (Med Associates Inc, Fairfax, Fairfax, VT, USA). Testing occurred either on day 1 (P→C) or on day 6 (C→P), 4 to 8 h after switching food. The test consisted of two trials: a sample trial and a test trial. During the sample trial, rats were allowed to freely explore two of the three arms, which had an identical floor color for 5 min; the third arm of the Y maze was kept inaccessible. After 1 h, a 5-min test trial was performed, in which the Y-maze was re-arranged as follows: (a) access to one of the two familiar arms was prevented; (b) the floor’s color of the other familiar arm was changed (familiar location-novel cue); (c) the arm, which was inaccessible during the sample trial, was made accessible and the color of its floor was the same than the floor’s color of the arms in the sample trial (novel location-familiar cue). Therefore, the “novelty” factor in a specific arm was a function of either its location (novel place) or the color of its floor (novel cue). Contextual cues surrounded the Y-maze. The floors’ color and the position of the arms (A, B, and C) were counterbalanced between subjects. The time spent exploring either the cue or place arm was measured and the % of time spent in the cue arm was used as the dependent variable and calculated as [(time spent in the cue arm)/(time spent in the cue arm + time spent in the place arm)] × 100. The subject’s preference for the novel cue arm is interpreted as a deficit in spatial learning or enhancement of stimulus-response learning; vice versa, the subject’s preference for the novel place arm is interpreted as an enhancement in spatial learning or a deficit in stimulus-response learning [27,30].

### 2.7. Spontaneous Alternation Behavior (SAB) Task

The Spontaneous Alternation Behavior (SAB) task probes spatial working memory and is based on the innate tendency of rodents to explore unexplored environments [31]. The apparatus consisted of a Y-shaped maze with three white, enclosed, opaque plastic arms (7.6 cm × 45.8 cm) at a 120° angle from each other (Med Associates Inc, Fairfax, VT, USA). Testing occurred either on day 1 (P→C) or on day 6 (C→P), 4 to 8 h after switching food. For testing, rats were placed in the center of the maze and were allowed to freely explore the three arms for 10 min (Figure 1B). Arm entry was considered when the entire body of the rat (except the tail) was within the arm. The number of entries and the number of alternations were recorded. Alternation was defined as entries into the three different arms on overlapping triad sets, a triad being a set of consecutive arm entries. Contextual cues surrounded the Y-maze. The percentage of spontaneous alternations was used as the dependent variable and was calculated as [(number of alternations)/(total arm entries-2)] × 100 [31]. Rodents typically prefer to investigate a new arm of the maze rather than return to one that was previously visited; a reduction of this tendency is interpreted as a deficit in spatial working memory.

### 2.8. Food Intake Experiments

For the evaluation of the effects of memantine on food intake, pre-weighed food was provided at the beginning of the dark cycle upon renewing access to the palatable diet (C→P phase), or to the chow diet (P→C phase) and weighed again 1 h after drug administration.

### 2.9. Perfusions and Immunohistochemistry

Following behavioral tests, rats were anesthetized and perfused 4–8 h after being switched either from the palatable diet to the chow diet (P→C phase) or from the chow diet to the palatable diet (C→P phase). Rats were transcardially perfused with Phosphate-Buffered Saline (PBS) 1X (pH = 7.4), followed by 4% paraformaldehyde buffered in Borax (pH = 9.5). Brains were dissected, post-fixed in ~20 mL of the same fixative for 24 h at 4 °C and then transferred to a 30% sucrose solution in PBS 1X at 4 °C until saturation. Brains were cut into 40 μm coronal sections using a cryostat and subsequently stored in a cryoprotectant solution at −20 °C until processed for immunohistochemistry. Microtubule associated protein doublecortin (DCX) expressed in immature cells (typically 2–3 weeks old adult newborn cells) was used as a sensitive marker of neurogenesis in the dentate gyrus [28,32]. Immunoreactivity of the endogenous cell cycle protein Ki-67 was used to determine the number of proliferating cells [33]. Every tenth section of the hippocampus (bregma range: −2.04 to −3.84) was stained with antisera raised against either DCX (goat polyclonal anti-DCX, 1:200, Santa Cruz Biotechnology, Dallas, TX, USA) or Ki-67 (mouse monoclonal anti-ki67, 1:100, Novocastra, UK). Immunochemistry was performed as previously described [28,34,35]. Briefly, free-floating sections were washed in 0.1M Tris-Buffered Saline (TBS), and endogenous peroxidase activity was quenched with 3% H_2_O_2_ for 10 min. Sections were incubated with a blocking solution (3% horse serum) at room temperature for 1 h, then in primary antibodies at 4 °C for 24 h, and then into secondary biotinylated antibodies (BA-9500, 1:200, and BA-2001, 1:400 Vector Laboratories, Burlingame, CA, USA for DCX and Ki-67, respectively) at room temperature for 1 h. Sections were then incubated in an avidin-biotin horseradish ABC solution (ABC kit, Vector Laboratories, Burlingame, CA, USA) in blocking solution for 1 h. DAB-H_2_O_2_ substrate was used to label antigenic sites according to the manufacturer’s instructions. Once the reaction was complete, sections were rinsed, mounted onto slides, allowed to dry before being dehydrated using graded alcohol concentrations, and cover-slipped using DPX mountant media (Electron Microscopy Sciences, Hatfield, PA, USA). The sections stained with Ki-67 were counterstained with cresyl violet to optimize visualization.

### 2.10. Quantification

Quantification of cell bodies was performed in accordance with the unbiased stereology approach. A parallel series of sections was analyzed for each staining batch. Brain sections were analyzed using an Olympus (Center Valley, PA, USA) BX-51 microscope equipped with a Rotiga 2000R live video camera (QImaging, Surrey, BC, Canada), a three-axis MAC6000 XYZ motorized stage (Ludl Electronics, Hawthorne, NY, USA) and a personal computer workstation. The dentate gyrus was outlined virtually on the digitized image of each section using the optical fractionator workflow module of Stereo Investigator software (MicroBrightField, Williston, VT, USA). One hemisphere was randomly chosen for each section; DG contours (including granular and subgranular layers) were drawn at low magnification using an Olympus PlanApo N 2X objective with numerical aperture 0.08 and counted using an Olympus UPlanFL N 40X objective with numerical aperture 0.75. All DCX + cells within the DG and all the Ki-67 + cells within 20 µm of the inner border of the granule cell layer (in the subgranular zone) were counted for each hemisphere. A guard zone of 2 μm and a dissector height of 20 μm were used. Immunostaining and mounting result in altered section thickness, which was, hence, measured at each counting site. An average section thickness was computed by the software and used to estimate the total volume of the sample region and total number of DCX + and Ki-67 + cells.

### 2.11. Statistical Analysis

Three-way mixed analyses of variance (ANOVAs) with Diet as a between-subjects factor and Week and Phase as within-subject factors were used to analyze the average daily food intake, the average body weight change, and the average feed efficiency. Two-way mixed analysis of variance (ANOVA) with Diet as a between-subjects factor and Week as a within-subject factor was used to analyze the cumulative food intake, the cumulative body weight gain, and the cumulative feed efficiency. Two-way analysis of variance (ANOVA) with Diet and Phase as between-subjects factors was used to analyze the memory tasks’ dependent variables as well as the DCX and the Ki-67 counting data. Two-way analysis of variance (ANOVA) with Diet and Dose as between-subjects factors was used to analyze the effects of memantine on the memory tasks. Two-way mixed analysis of variance (ANOVA) with Diet as a between-subjects factor and Dose as a within-subject factor was used to analyze the effects of memantine on food intake. Following significant effect of ANOVAs (*p* ≤ 0.05), Duncan post-hoc tests were performed. Data analysis and graph processing were performed using Statistica 7 (StatSoft, Tulsa, OK, USA) and SigmaPlot 12.0 (Systat Software Inc., Chicago, IL, USA) software, respectively.

## 3. Results

### 3.1. Effects of Palatable Diet Alternation on Food Intake and Body Weight

Extended intermittent access to palatable food led to progressive undereating of standard chow diet when palatable food was withdrawn, as well as to overeating of palatable food upon renewed access (Diet × Week × Phase: *F*_(8,296)_ = 24.39, *p* ≤ 0.0001; Figure 1B). During the first cycle, Chow/Palatable rats hyperphagia of the palatable diet resulted in a higher cumulative caloric consumption than controls; however, the progressive amplification of Chow/Palatable rats’ hypophagia, observed during the next C phases, led to a significantly smaller cumulative intake starting from week 6 (Diet × Week, *F*_(8,296)_ = 6.70, *p* ≤ 0.0001; Figure 1C). With diet cycling, Chow/Palatable rats also showed amplified cycling of local body weight change in contrast to the neutral weight trajectory of Chow/Chow rats (Diet × Week × Phase: *F*_(8,296)_ = 18.39, *p* ≤ 0.0001, Supplementary Figure S1A). These body weight changes across the phases were not entirely explained by feeding changes, as demonstrated by the significant differences in local feed efficiency between the two groups (Diet × Week × Phase: *F*_(8,296)_ = 16.59, *p* ≤ 0.0001; Supplementary Figure S1C). A main interaction between Diet, Schedule, and Week was observed in the cumulative body weight gain (Diet × Week, *F*_(8,296)_ = 4.00, *p* ≤ 0.0001, Supplementary Figure S1B), likely resulting from a slow, constant tendency of Chow/Palatable diet rats to gain more weight than Chow/Chow control rats weekly; however, post-hoc group comparisons did not reveal any reliable difference in body weight change up to the ninth week of diet cycling between groups. No difference in cumulative feed efficiency between groups was observed (Diet × Week, *F*_(8,296)_ = 0.94, *n.s.*, Supplementary Figure S1D).

### 3.2. Effects of Palatable Diet Alternation on Spatial Memory Using the Object Location (OL) Task

In the OL task, palatable diet cycled rats showed a withdrawal-dependent deficit in spatial memory (Diet: *F*_(1,33)_ = 3.66, *n.s.*; Phase: *F*_(1,33)_ = 2.92, *n.s.*; Diet × Phase: *F*_(1,33)_ = 4.76, *p* ≤ 0.05; Figure 2A). Control Chow/Chow rats showed greater exploration of the novel location compared to chance in both C (*p* ≤ 0.05) and P phase (*p* ≤ 0.05). However, Chow/Palatable rats, during withdrawal from the palatable diet, showed impairment in recognizing the novel location of the familiar object as compared to both Chow/Chow rats in C Phase and Chow/Palatable rats in the P Phase. Accordingly, palatable food-withdrawn Chow/Palatable rats explored similarly the two objects as revealed by a not statistically significant difference from chance (*p* > 0.05). Conversely, Chow/Palatable rats showed greater exploration of the novel location compared to chance (*p* ≤ 0.05) and did not differ from controls (Figure 2A).

### 3.3. Effects of Palatable Diet Alternation on Recognition Memory Using the Novel Object Recognition (NOR) Task

In the NOR task, palatable diet cycled rats did not perform differently than the controls in recognition memory (Diet: *F*_(1,35)_ = 0.03, *n.s.*; Phase: *F*_(1,35)_ = 0.91, *n.s.*; Diet × Phase: *F*_(1,35)_ = 0.02, *n.s.*; Figure 2B). Control Chow/Chow rats showed greater exploration of the novel location compared to chance in both C (*p* ≤ 0.002) and P phase (*p* ≤ 0.02). Similarly, Chow/Palatable rats showed greater exploration of the novel location compared to chance in both C (*p* ≤ 0.002) and P phase (*p* ≤ 0.05). Chow/Palatable rats during both C and P phase did not differ from controls (Figure 2B).

### 3.4. Effects of Palatable Diet Alternation on the Preference for Stimulus—vs. Spatial—Response Learning Using the Novel Cue vs. Novel Place Preference (NC-NP Preference) Task

In the NC-NP Preference task, palatable diet cycled rats showed a withdrawal-dependent preference for stimulus-response learning (Diet: *F*_(1,25)_ = 5.22, *p* ≤ 0.05; Phase: *F*_(1,25)_ = 6.89, *p* ≤ 0.02; Diet × Phase: *F*_(1,25)_ = 0.34, *n.s.*). Control Chow/Chow rats did not show any preference with the novel cue vs. the novel place compared to chance in both C (*p* > 0.05) and P phase (*p* > 0.05). However, Chow/Palatable rats, during withdrawal from the palatable diet, showed a preference for the novel cue as compared to both Chow/Chow rats in C Phase and Chow/Palatable rats in the P Phase. Accordingly, palatable food-withdrawn Chow/Palatable rats showed greater exploration of the novel cue compared to chance (*p* ≤ 0.001). Conversely, Chow/Palatable rats showed a similar preference for the novel cue vs. the place compared to chance (*p* > 0.05) and did not differ from controls (Figure 2C).

### 3.5. Effects of Palatable Diet Alternation on Working Memory Using the Spontaneous Alternation Behavior (SAB) Task

In the SAB task, palatable diet cycled rats did not perform differently than the controls in working memory (Diet: *F*_(1,35)_ = 0.01, *n.s.*; Phase: *F*_(1,35)_ = 0.01, *n.s.*; Diet × Phase: *F*_(1,35)_ = 0.77, *n.s.*). Control Chow/Chow rats performed a greater number of spontaneous alternations compared to chance in both C (*p* ≤ 0.001) and P phase (*p* ≤ 0.001). Similarly, Chow/Palatable rats performed a greater number of spontaneous alternations compared to chance in both C (*p* ≤ 0.001) and P phase (*p* ≤ 0.001). Chow/Palatable rats during both C and P phase did not differ from the controls (Figure 2D).

### 3.6. Effects of Palatable Diet Alternation on Cell Proliferation and Neurogenesis in the Dentate Gyrus of the Hippocampus

**Figure 3:**
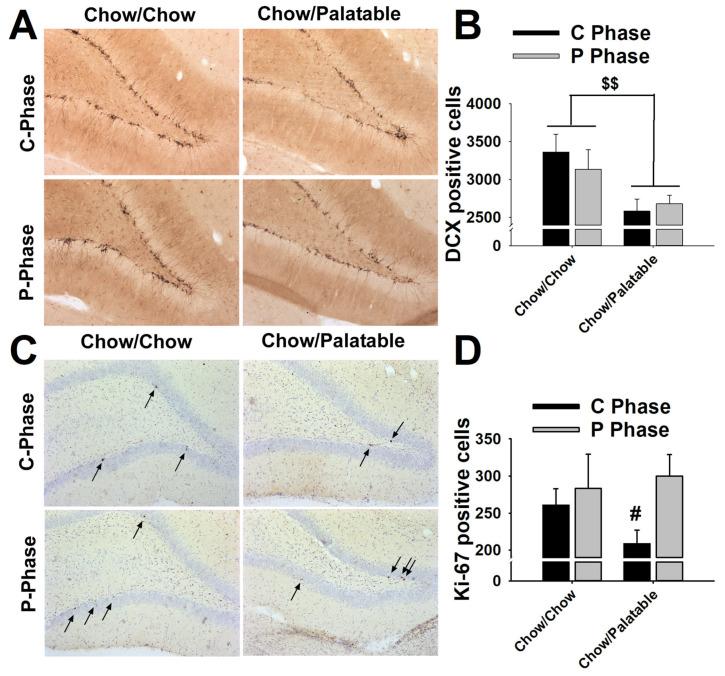
Effects of repeated, alternating 5-day access to chow and 2-day access to either regular chow (Chow/Chow) or a palatable diet (Chow/Palatable) in male Wistar rats on (**A**,**B**) doublecortin (DCX) and (**C**,**D**) Ki-67 expression, in the dentate gyrus of the dorsal hippocampus. (**A**,**C**) Representative micrographs of DCX and Ki-67 immunoreactivity in the in the dentate gyrus of the dorsal hippocampus. (**B**,**C**) (*n* = 7–10/group) Data show *M* ± SEM. $$ *p* ≤ 0.01 main effect of Diet; # *p* ≤ 0.05, Chow/Palatable in C Phase vs. Chow/Palatable in P phase.

Irrespective of diet phase, intermittent access to the palatable diet significantly reduced the expression of DCX + cells in the dentate gyrus of the hippocampus of Chow/Palatable rats as compared to the controls (Diet: *F*_(1,31)_ = 9.56, *p* ≤ 0.005; Phase: *F*_(1,31)_ = 0.11, *n.s.*; Diet × Phase: *F*_(1,31)_ = 0.67, *n.s.*; Figure 3A,B).

Interestingly, Chow/Palatable rats, when withdrawn from the palatable diet, showed a significant reduction in Ki-67 + cells in the dentate gyrus of the hippocampus as compared to palatable food-refed diet cycled rats (Diet: *F*_(1,30)_ = 0.38, *n.s.*; Phase: *F*_(1,30)_ = 4.00, *p* ≤ 0.05; Diet × Phase: *F*_(1,30)_ = 1.46, *n.s.*; Figure 3C,D).

### 3.7. Effects of Memantine Treatment on Spatial Memory in Diet Cycled Rats

**Figure 4:**
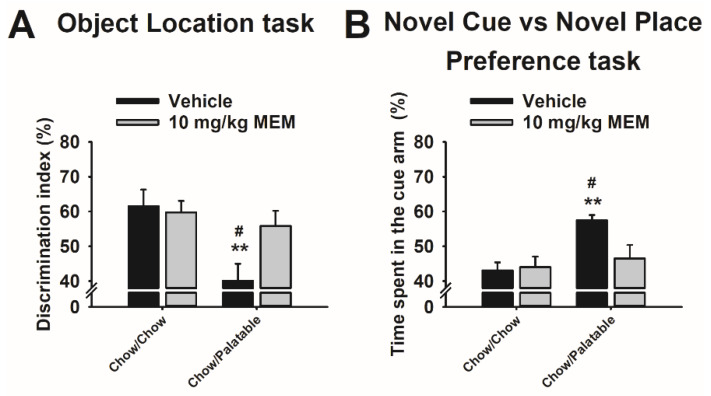
Effects of systemic administration of memantine (0, 10 mg/kg) on (**A**) Object Place Recognition task and (**B**) Novel Cue vs. Novel Place Preference (NC-NP Preference) task during the C Phase of Chow/Chow and Chow/Palatable male Wistar rats undergoing the ad libitum diet alternation (*n* = 6–10/group). Data show *M*±SEM. # *p* ≤ 0.05, Chow/Palatable in C phase vs. Chow/Palatable in P phase; ** *p* ≤ 0.01 Chow/Palatable in C phase vs. Chow/Chow in C phase.

Memantine reversed the withdrawal-dependent deficit in spatial memory in palatable diet cycled rats (Diet: *F_(_*_1,35)_ = 8.44, *p* ≤ 0.01; Treatment: *F*_(1,35)_ = 2.53, *n.s.*; Diet × Dose: *F*_(1,35)_ = 3.98 *p* ≤ 0.05; Figure 4A). As observed in our previous experiment, vehicle-treated Chow/Palatable rats showed an impairment recognizing the novel location of the object during the C phase, and this effect was fully prevented by pretreatment with memantine. Memantine pretreatment did not affect the performance of Chow/Chow rats in this test.

### 3.8. Effects of Memantine Treatment on the Preference for Stimulus—vs. Spatial—Response Learning in Diet Cycled Rats

Memantine reversed the withdrawal-dependent preference for stimulus-response learning in palatable diet cycled rats (Diet, *F_(_*_1,28)_ = 7.67, *p* ≤ 0.01; Treatment, *F*_(1,28)_ = 2.70, *n.s.*; Diet × Dose, *F*_(1,28)_ = 3.84 *p* = 0.059; Figure 4B). As observed in our previous experiment, vehicle-treated Chow/Palatable rats showed a preference for stimulus-response learning during the C phase, and this effect was fully prevented by pretreatment with memantine. Memantine pretreatment did not affect the performance of Chow/Chow rats in this test.

### 3.9. Effects of Memantine on Food Intake and Body Weight of Diet Cycled Rats

**Figure 5:**
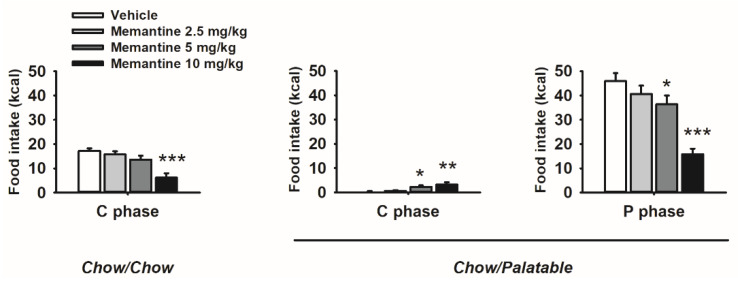
Effects of systemic administration of memantine (0–10 mg/kg) on food intake in Chow/Chow and Chow/Palatable male Wistar rats undergoing the ad libitum diet alternation. (*n* = 10/group) Data show *M* ± SEM. * *p* ≤ 0.05, ** *p* ≤ 0.01, *** *p* ≤ 0.001 vs. vehicle.

Memantine differentially affected food intake as a function of food history, and diet provided resulting in a strong interaction effect among factors (Diet × Dose, *F*_(9,81)_ = 15.01, *p* ≤ 0.00001; Figure 5). Specifically, drug treatment dose-dependently decreased palatable diet intake and increased chow food intake in Chow/Palatable rats, while it decreased standard chow intake in Chow/Chow control rats. Interestingly, memantine effects were more potent in palatable diet rats: standard chow intake in Chow/Chow rats decreased only at the highest dose of memantine injected (10 mg/kg), whereas both the 5 and the 10 mg/kg doses were able to either decrease the palatable diet intake or increase the standard diet intake in Chow/Palatable rats.

## 4. Discussion

The major findings of the present study were the following: (1) withdrawal from intermittent access to palatable food is accompanied by hippocampus-dependent memory function impairment; (2) deficit in memory function is associated with disruption of dentate gyrus neurogenesis in rats exposed to intermittent access to palatable food; (3) the uncompetitive NMDAR antagonist, memantine, fully rescues the memory function impairment in rats withdrawn from intermittent access to palatable food; (4) memantine fully blocks excessive intake of palatable food and ameliorates hypophagia of the standard diet in cycled rats.

In this study, we found that withdrawal from a highly palatable diet was responsible for memory function impairment. Notably, the deficit in memory function observed in palatable food-withdrawn rats was selective for hippocampus-dependent memory function, as revealed by both the inability of cycled rats to recognize the novel location of a familiar object, and the preference for a stimulus-response at the expenses of spatial learning. Indeed, an intact hippocampus is required in the mnemonic functions that allow the recognition of a novel position of a specific object [36], as well as the recognition of a novel contextual environment over a novel cue [27,30]. Conversely, palatable food-withdrawn rats, when tested in an NOR task, showed an intact recognition memory, as shown by an increased spontaneous tendency to explore a novel object over a familiar one, a memory function that is dependent on extra-hippocampal cortices (e.g., perirhinal cortex) [37]. Similarly, when diet cycled rats were withdrawn from the palatable diet and tested in the SAB task, no deficits in working memory were observed, a memory function that is mainly dependent on the prefrontal cortex [38].

Memory impairment was observed when rats were withdrawn from the highly palatable diet (i.e., during the C phase), but not when access to the palatable diet was renewed (i.e., during the P Phase). We can confidently exclude that the observed memory and neurogenesis deficits are due to the negative energy homeostatic balance resulting from the hypophagia of the standard diet during palatable food withdrawal. Indeed, caloric restriction has been extensively shown to produce a beneficial effect to both memory function and neurogenesis (see review [39]). Instead, according to a negatively reinforced mechanism, a more plausible explanation for the observed results is that the stressful emotional state induced by palatable food withdrawal is responsible for the observed memory deficit. Indeed, we have previously shown that, in rats undergoing similar diet cycling procedure, withdrawal from palatable food is accompanied by the emergence of a negative affect characterized by anxiety-like and depressive-like behaviors, similar to what observed in drug addiction [15,16,20,23,40]. Accordingly, stress has been extensively shown to negatively impact mnemonic processes [41], facilitating dorsal striatum-dependent “habit” memory at the expense of hippocampus-dependent “cognitive” memory [42].

The present results are in line with human studies showing that dietary factors are associated with the emergence of hippocampal dysfunction and worse hippocampal-dependent memory performance [43,44,45,46]. Interestingly, previous studies have shown that exposure to energy-dense diets impairs place, but not object, recognition memory in rats [47,48]. Our results provide evidence of a differential role for palatable food withdrawal and refeeding in spatial memory performance in rats undergoing a diet alternation procedure.

Hippocampal neurogenesis has been shown to critically support spatial learning and memory processing [49]. Indeed, specific knockdown of adult neurogenesis results in an impairment of both spatial and object recognition memory in adult rats [50]. Consistent with a hippocampal-dependent memory deficit, dentate gyrus neurogenesis in cycling rats was disrupted as compared to rats, which were instead monotonously fed the standard chow diet. Interestingly, while the reduction in proliferating cells was diet phase-dependent, as observed by a selective reduction in Ki-67 in palatable food withdrawal, newborn neuron disruption was diet phase-independent, as measured by an overall decrease of DCX in both C and P phases. A possible explanation for these apparently incongruent effects observed in proliferating cells and newborn neurons in the two diet phases may be related to the temporal dynamics of the different stages of the neurogenic process. Cell proliferation in SGZ is a relatively short phenomenon (~1 day, [51]) that precedes the differentiation into young immature neurons [12], a stage that instead can last up to four weeks [52]. Not surprisingly, the number of proliferating cells correlates with the number of newborn immature neurons [32]. Therefore, in the conditions of these experiments, Ki-67 measurement represents a snapshot of how many new cells were proliferating in a specific diet-phase, while DCX measurement reflects the number of newborn neurons within the previous few weeks of diet cycling. Therefore, since stress is known to be an extrinsic negative modulator of cell proliferation [12], it is plausible to assume that while every stressful palatable food withdrawal event negatively affected the number of proliferating cells in that specific phase, it also decreases the overall number of newborn neurons, irrespective of the diet phase.

Eating disorders generally occur more frequently in women than in men [53]. In addition, women appear to be at greater risk to develop affective and stress-related disorders following traumatic experiences, from adolescence throughout adulthood [54]. Moreover, it has been shown that a gender difference in spatial navigation exists, with women typically performing worse than males [55,56]. Although the animal model of diet alternation used here has been developed in both female and male rats, no direct sex comparisons have ever been performed [15,16,20,21,22,23]. Future studies will be important to make direct comparisons between sexes in the consummatory, stress-related, learning and memory outcomes following this diet alternation procedure.

Interestingly, there is evidence suggesting that hippocampal pathology is associated with the onset of increased food intake and body weight gain. Indeed, damage in the hippocampus has been related with a reduced ability to inhibit caloric intake and ultimately, to increased body weight [9]. Moreover, selective hippocampal lesions as well as neurodegenerative diseases affecting this brain structure are often associated with increased energy intake [9]. Therefore, while the present data do not allow for any causal relationship between feeding and altered hippocampal function induced by diet alternation, we speculate that hippocampal damage may also contribute to the excessive intake of palatable diet observed here. Once again, we can confidently exclude that the observed disruption of hippocampal neurogenesis process is due to the energy deprivation as caloric restriction has been consistently shown to increase hippocampal neurogenesis [39].

Our results show that treatment with the uncompetitive NMDAR uncompetitive antagonist memantine was able to fully restore the mnemonic deficit in rats withdrawn from the palatable diet. In addition, memantine both blocked the excessive intake of palatable food and ameliorated the withdrawal-induced hypophagia of the otherwise acceptable standard diet in rats undergoing the palatable diet alternation procedure. Therefore, the same drug was able to improve both the mnemonic and consummatory maladaptive behavioral responses to palatable diet alternation. Memantine is an FDA approved medication that ameliorates mnemonic symptoms in patients with moderate and severe disease, and has also proved to be effective in individuals affected by binge eating disorders [57]. Indeed, memantine has been shown to reduce the frequency of binging days and episodes, the severity of the eating disorder in humans [58,59], and forms of excessive eating in animals [19,60,61]. Here, we provide evidence that memantine can be a potential treatment option for both memory deficits and aberrant feeding patterns induced by the same diet alternation procedure. Future studies will be helpful to determine whether memantine treatment in this animal model can also reverse impairment in neurogenesis.

In summary, the present study shows that a pattern of palatable food withdrawal dieting/overeating leads to a significant impairment in hippocampal-dependent tasks paralleled by a decrease in hippocampal neurogenesis expressed as a reduced number of immature neurons in the dentate gyrus and a reduction in progenitor cells proliferation. Remarkably, treatment with the uncompetitive NMDAR antagonist memantine proved effective in preventing the phase-specific learning and memory deficits as well as in both reducing the exacerbated binge-like eating when palatable food was presented, and attenuating withdrawal-induced hypophagia of regular food. The present data supports the hypothesis that a history of repeated discrete alternations in food palatability across time profoundly impacts hippocampal function and plasticity, which may in turn facilitate aberrant feeding behavior. Moreover, this study provides evidence that supports the use of memantine as a potential pharmacological treatment for mnemonic and consummatory symptomatology in disorders of eating behavior.

## Supplementary Materials

The following are available online at https://www.mdpi.com/2072-6643/12/5/1520/s1, Figure S1: Effects of repeated, alternating 5-day access to chow and 2-day access to either chow 13 (Chow/Chow) or highly preferred chocolate-flavored sugary diet (Chow/Palatable) on (**A**) average body weight change, (**B**) cumulative body weight gain, (**C**) Average feed efficiency, and (**D**) cumulative feed efficiency, (*n* = 19–20/group). Data show M ± SEM. * *p* ≤ 0.05, ** *p* ≤ 0.01, *** *p* ≤ 0.001 vs. Chow/Chow.
