# Supplementary material for: Withdrawal from Extended, Intermittent Access to A Highly Palatable Diet Impairs Hippocampal Memory Function and Neurogenesis: Effects of Memantine
Source: Nutrients. 2020 May 23;12(5):1520. doi: 10.3390/nu12051520 (PMC7284648; doi:10.3390/nu12051520)
Supplement: Supplementary file 1 [file nutrients-12-01520-s001.pdf]

nutrients

Research Manuscript

Withdrawal from extended, intermittent access to a highly palatable diet impairs hippocampal memory function and neurogenesis: effects of memantine

Antonio Ferragud [1], Clara Velázquez-Sánchez [1], Ali Al Abdullatif [1], Valentina Sabino [1] and Pietro Cottone [1,*]

[1] Laboratory of Addictive Disorders, Departments of Pharmacology and Psychiatry, Boston University School of Medicine, Boston, MA, USA

* Correspondence: cottone@bu.edu; 001-617-358-1950

Supplementary Figure S1

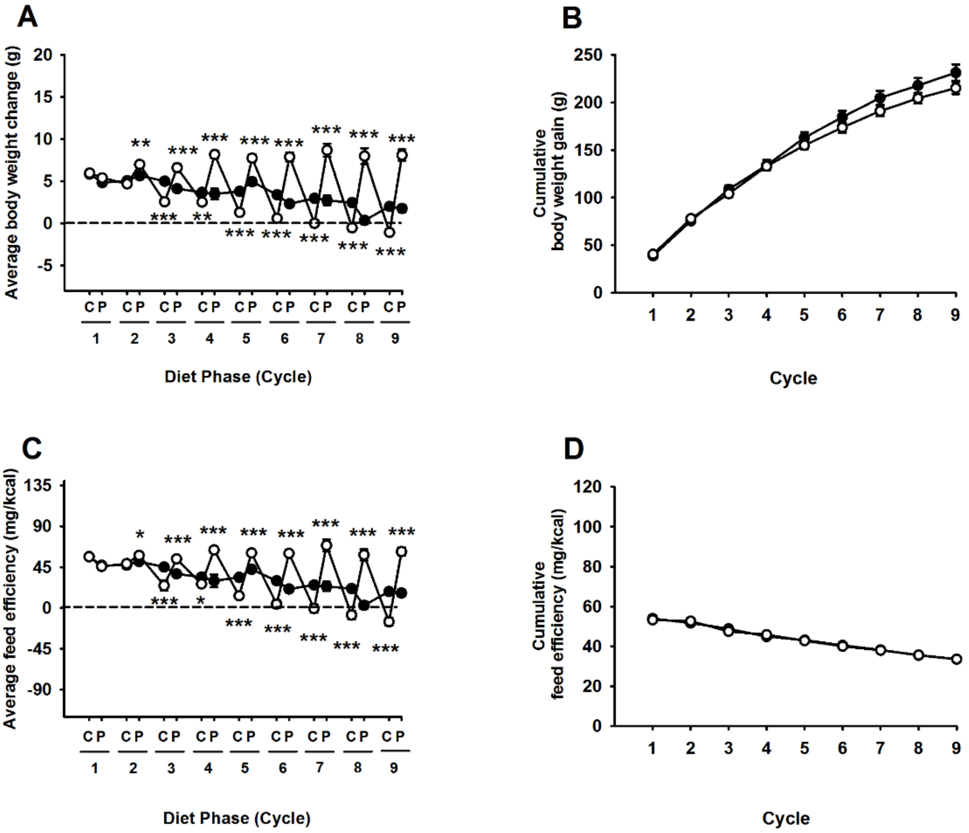

Figure 5. Effects of repeated, alternating 5-day access to chow and 2-day access to either chow (*Chow/Chow*) or highly preferred chocolate-flavored sugary diet (*Chow/Palatable*) on **(A)** average body weight change, **(B)** cumulative body weight gain, **(C)** Average feed efficiency, and **(D)** cumulative feed efficiency, (*n*=19-20/group). Data show *M*±SEM. * $p \leq 0.05$, ** $p \leq 0.01$, *** $p \leq 0.001$ vs. *Chow/Chow*.
